# Effect of remimazolam versus propofol for the induction of general anesthesia on cerebral blood flow and oxygen saturation in elderly patients undergoing carotid endarterectomy

**DOI:** 10.1186/s12871-023-02095-z

**Published:** 2023-05-04

**Authors:** Jianling Gao, Chengdi Yang, Qiuyuan Ji, Jian Li

**Affiliations:** 1 Department of Critical Care Medicine, Dushu Lake Hospital Affiliated to Soochow University, Suzhou, Jiangsu China; 2 Department of Critical Care Medicine, The First Affiliated Hospital of Soochow University, Suzhou, Jiangsu China; 3 Department of Anesthesiology, Dushu Lake Hospital Affiliated to Soochow University, Suzhou, Jiangsu China; 4 Department of Anesthesiology, The First Affiliated Hospital of Soochow University, Suzhou, Jiangsu China

**Keywords:** Remimazolam, Propofol, Anesthesia induction, Cerebral oxygen saturation, Carotid endarterectomy

## Abstract

**Background:**

This study was conducted to investigate the effects and safety of remimazolam versus propofol on cerebral oxygen saturation and cerebral hemodynamics during the induction of general anesthesia in patients receiving carotid endarterectomy (CEA), so as to provide theoretical basis for better clinical application of remimazolam.

**Methods:**

Forty-three patients (60–75 years old) with carotid artery stenosis (carotid artery stenosis greater than 70%) were randomly divided into the remimazolam group (R group) and the propofol group (P group). Anesthesia was induced with remimazolam (0.3 mg/kg) or propofol (1.5-2 mg/kg) individually. At time of admission (T0), post-anesthesia induction (T1), consciousness disappears (T2), 1 min after loss of consciousness (T3), 2 min after loss of consciousness (T4) and pre-endotracheal intubation (T5), measurement in patients with regional cerebral oxygen saturation (SrO_2_), average blood flow velocity (Vm), resistance index (RI), mean arterial pressure (MAP), heart rate (HR) and cardiac index (CI) were recorded.

**Results:**

SrO_2_ increased significantly in both groups after induction of anesthesia compared with baseline (*P* < 0.05) and decreased after loss of consciousness (*P* < 0.05). There was no difference in the mean value of the relative changes in SrO_2_ between the groups. Meanwhile, the Vm, RI, HR and CI of each time point between two groups showed no statistically significant difference (*P* > 0.05) while MAP in group P at T5 was lower than that in group R individually(*P* < 0.05). In each group, Vm, HR, CI and MAP at T2-T5 were all significantly reduced compared with T1, with statistically differences(*P* < 0.05). Specifically, there was no difference of RI at each time between or within groups(*P* > 0.05).

**Conclusions:**

Our study revealed that remimazolam can be administered safely and effectively during the induction of general anesthesia for carotid endarterectomy in elder population and it demonstrated superiority in hemodynamic changes compared with propofol.

**Clinical trial registration:**

This trial was retrospectively registered with the Chinese Clinical Trial Registry. Registration number: ChiCTR2300070370. Date of registration: April 11, 2023.

## Introduction

Carotid artery stenosis is a major reason of stroke in elderly, accounting for about 20% of all cases and leads to high mortality and heavy economic burden [1, 2]. Carotid endarterectomy (CEA) remains the recommended treatment for the prevention of stroke in symptomatic patients with carotid artery stenosis, which is usually performed under general anesthesia [3, 4]. Most of patients are elderly accompanied with cardiovascular diseases, so severe hemodynamic fluctuations are easy to occur during the induction period of general anesthesia, which may not only exacerbate the preoperative cerebral blood supply deficiency, but also cause cerebral hypoxia and thereby lead to postoperative nervous system function deficiency [5, 6]. Therefore, for patients with severe carotid artery stenosis, due to the impairment of carotid pressure receptors and cerebral blood flow automatic regulation, choosing anesthetics which has less adverse effects and protects from cerebral ischemia or hypoxia is necessary.

Cerebral oxygen saturation (SrO_2_) can reflect the balance of oxygen supply and demand of brain tissue, which is related to the arterial oxygen saturation, hemoglobin, cerebral blood flow (CBF) and cerebral metabolic rate of oxygen (CMRO_2_). Thus, monitoring SrO_2_ is a noninvasive method to observe the changes in CBF and predict the occurrence of cerebral ischemia during the induction of general anesthesia [7]. Meanwhile, transcranial doppler (TCD) can measure mean blood velocity (Vm) and resistance index (RI) of the middle cerebral artery. Thus, we are able to investigate cerebral blood flow and oxygen saturation using these indexes.

Propofol is frequently used intravenously for the induction of sedation during general anesthesia, which is often associated with hemodynamic depression. Previous studies have shown that anesthesia induction with propofol decreased the CBF as CMRO_2_ was reduced due to the different degrees of blood pressure decline [8], and may break the cerebral oxygen balance. In addition, there were gender differences in the effect of propofol anesthesia on perioperative cerebral oxygenation under general anesthesia in patients undergoing CEA, female patients had significantly lower regional cerebral oxygen saturation values compared with male patients during clamping of the carotid artery [9]. Remimazolam is an ultrashort-acting benzodiazepine inducing sedation with rapid onset, good control of the depth of anesthesia, full and predictable recovery, a benign safety profile particularly in terms of hemodynamic effects [10]. According to the latest researches, remimazolam provided adequate procedural sedation for endoscopy and its actual anesthetic effect is not inferior to that of traditional anesthesia-inducing drugs [11]. Remimazolam has the same efficacy as propofol in induction and maintenance of general anesthesia [12]. However, the use of remimazolam for induction of general anesthesia in patients undergoing carotid endarterectomy has not been reported.

Hence, we tested the hypothesis that remimazolam will maintain CBF and SrO_2_ better than propofol in terms of carotid endarterectomy as a sedative for the induction of general anesthesia. To validate this, we compared the SrO_2_ and cerebral hemodynamics during the anesthesia induction.

## Methods

### Study design

**Fig. 1:**
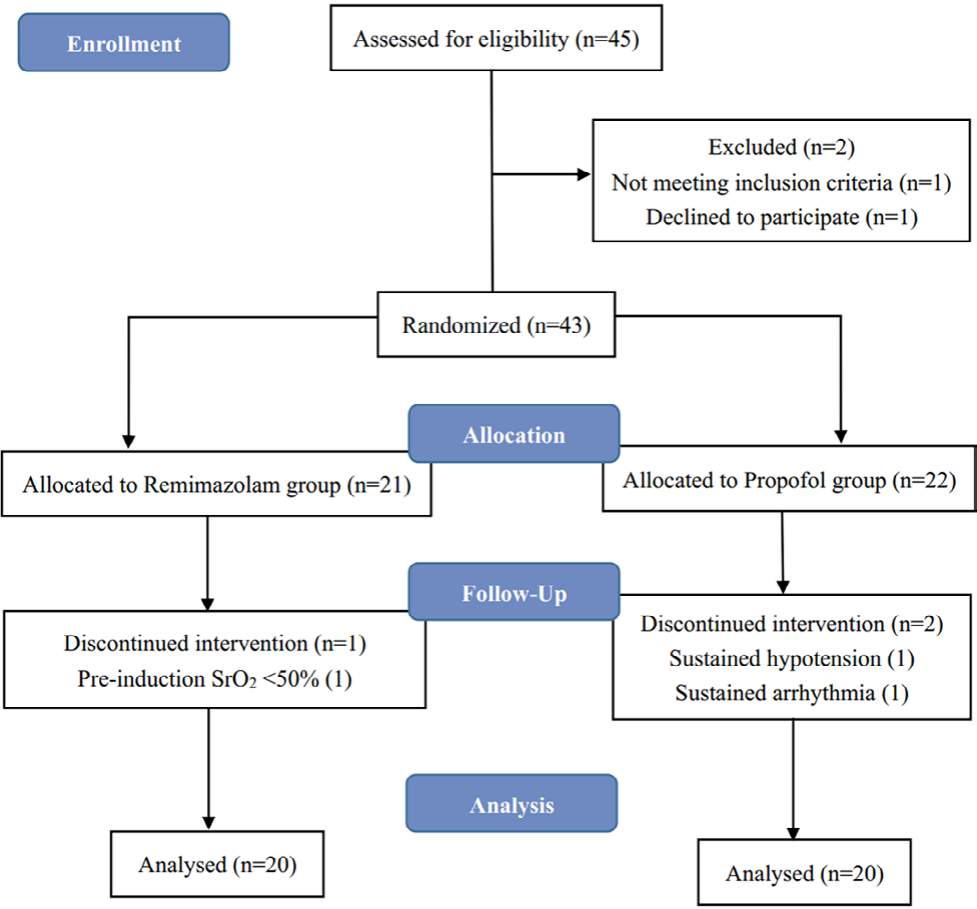
Enrolment flow diagram

This randomized clinical trial was approved by the Institutional Review Board of The Dushu Lake Hospital Affiliated to Soochow University (Fig. 1), all methods were carried out in accordance with relevant guidelines and regulations. 43 Patients (average age 67.20 ± 4.29) scheduled to undergo CEA under general anesthesia were randomly allocated into the propofol group or remimazolam group using Excel program RAND function. Patients excluded from this study included mentally disabled patients, patients with ASA class ≥ V, alcohol or drug abusers, patients being administered drugs that may affect study considered by researchers. The indications for CEA in this study are symptomatic patients with stenosis > 70% confirmed by ultrasound and all CEAs performed in this study were primary repairs.

### Experimental procedure

After patient arriving in the operating room, 5-lead electrocardiography, pulse oximetry, and non-invasive automatic blood pressure cuff were implemented for basic monitoring. INVOS cerebral oximeter (Somanetics, Troy, MI, USA) was applied for monitoring of operative side SrO_2_. To assess the depth of anesthesia, BIS® A-2000™ (Aspect Medical Systems, Natick, MA, USA) was attached to the contralateral forehead. A 20-gauge catheter was placed in the radial artery after local anesthetic infiltration to monitor continuous arterial blood pressure and CI (Cardiac Index) by using Flotrac/vigileo system (Edwards Lifesciences, Irvine, Ca, USA). Meanwhile, ultrasonic probe was placed on the temporal window with frequency set at 2 MHz and depth set at 50-55 mm to monitor the cerebral blood flow velocity.

The induction of general anesthesia was performed via infusion of 0.3 mg/kg remimazolam in R group or 1.5-2 mg/kg propofol in P group within 30 s [13, 14]. Additional dose was given with 2.5 mg remimazolam or 50 mg propofol if the BIS > 60 after 3 min. When adequate sedation (BIS = < 60) was achieved and confirmation of the loss of consciousness, sufentanil(3ug/kg) and cis-atracurium(2 mg/kg) was given to fulfill endotracheal intubation. General anesthesia was maintained by sevoflurane by adjust concentration to achieve a BIS value of 40–60.

### Cerebral oxygen saturation and hemodynamics record

This study set several time points to record index during the whole process of the induction of anesthesia in both groups. Specifically, after admission of patient, the basal levels of SrO_2_, MAP, HR, CI, RI and Vm were recorded as T0. Data were recorded simultaneously and continuously after induction of anesthesia(T1), loss of consciousness(T2), every 1 min for 2 min after loss of consciousness(T3-T4) and before intubation(T5).

### Statistical analysis

Previous studies [15]in patients receiving CEA showed maximal decreases in SrO_2_ of 8.1% ± 6.2% after induction in the propofol group. Therefore, assuming maximal decreases in SrO_2_ of 3% in remimazolam group, the sample size was calculated to show the difference between groups and 18 patients per group were required to achieve adequate power (*P* = 0.05; β = 0.8) to detect a difference. Patients’ characteristics, intraoperative variables and indexes between groups were analyzed using Student’s t-test or Pearson’s chi-square test. Continuous variables, including SrO_2_, blood pressure and heart rate, were analyzed by repeated measures analysis of variance (ANOVA) within each group. Comparisons between groups at each time point were made using two-tailed unpaired t-test. Statistical analysis was performed using the IBM SPSS Statistics version 19 (IBM, Chicago, IL, USA). All data are expressed as means ± standard deviation and *P* < 0.05 was considered to be statistically significant.

## Results

A total of 43 patients were included initially and randomized 21 to remimazolam group, 22 to propofol group. Among these patients, one patient of the remimazolam group with low SrO_2_ (pre-induction basal value < 50%) and 2 patients of the propofol group that showed sustained hypotension or arrhythmia intraoperatively were excluded. patients’ characteristics including gender, weight, height and BMI were similar in both groups (Table 1). Meanwhile, there were no significant differences in intraoperative values of degree of carotid stenosis, total anesthesia time and total operation time between the groups (Table 2).

**Table 1:** Patients Characteristics in groups

	Remimazolam(n = 20)	Propofol(n = 20)	*P* value
Age(yrs)	67.18 ± 4.38	67.21 ± 4.42	0.946
Sex			0.749
Male	16(80.0%)	15(75.0%)	
Female	4(20.0%)	5(25.0%)	
Weight(kg)	62.92 ± 11.38	61.42 ± 11.29	0.518
BMI(kg/cm^2^)	23.46 ± 2.75	23.10 ± 2.91	0.668
ASA grade			0.519
II	7(35.0%)	9(45.0%)	
III	13(65.0%)	11(55.0%)	
Cigarette			0.337
Yes	13(65.0%)	10(50.0%)	
No	7(35.0%)	10(50.0%)	

Values are mean ± SD or numbers

### Values are mean ± SD or numbers

**Table 2:** Intraoperative Variables in groups

	Remimazolam(n = 20)	Propofol(n = 20)	*P* value
Carotid stenosis (%)	75.32 ± 5.13	76.25 ± 5.52	0.719
Time to loss of consciousness(s)	64.13 ± 9.28	33.46 ± 7.15	0.001
Total anesthesia time(min)	136.82 ± 18.37	136.72 ± 19.82	0.982
Total operation time(min)	116.38 ± 18.47	115.57 ± 18.73	0.780
Awakening time(min)	15.83 ± 2.72	16.95 ± 3.32	0.973
Cardio-cerebrovascular accident	0	0	/

Values are mean ± SD or numbers

### SrO_2_ changes in groups

**Fig. 2:**
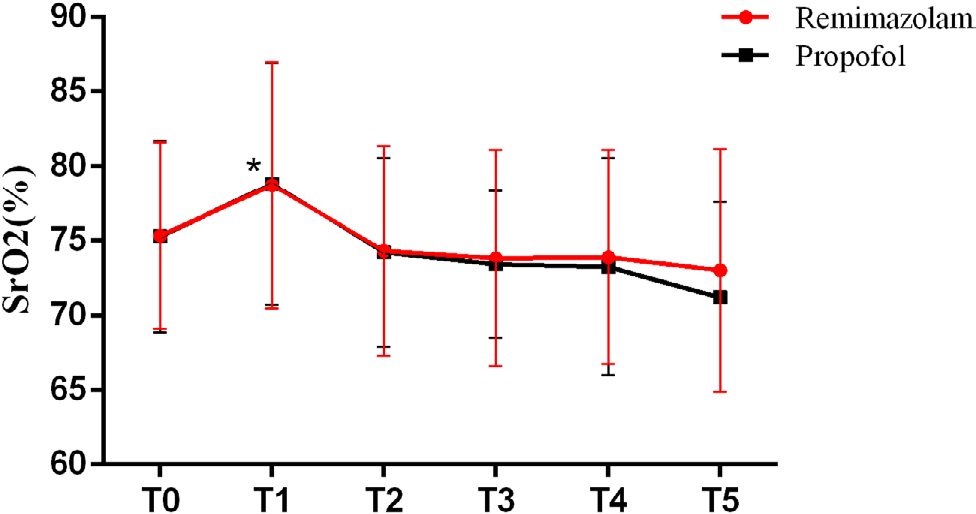
Cerebral oxygen saturation (SrO_2_) changes at post-induction of anesthesia(T1), loss of consciousness(T2), every 1 min for 2 min after loss of consciousness(T3-T4) and pre-intubation(T5). Data are expressed as mean ± standard deviation. *P < 0.05 vs. Baseline in each group

SrO_2_ increased significantly at T1 in both groups compared with baseline(T0) in remimazolam group and propofol group(*P* < 0.05). However, there was no difference in the change of SrO_2_ between remimazolam group and propofol group(*P* > 0.05) at each time point from T1-T5. Each group showed a downstream trend from T2-T5 (Fig. 2).

### CBF changes in groups

**Fig. 3:**
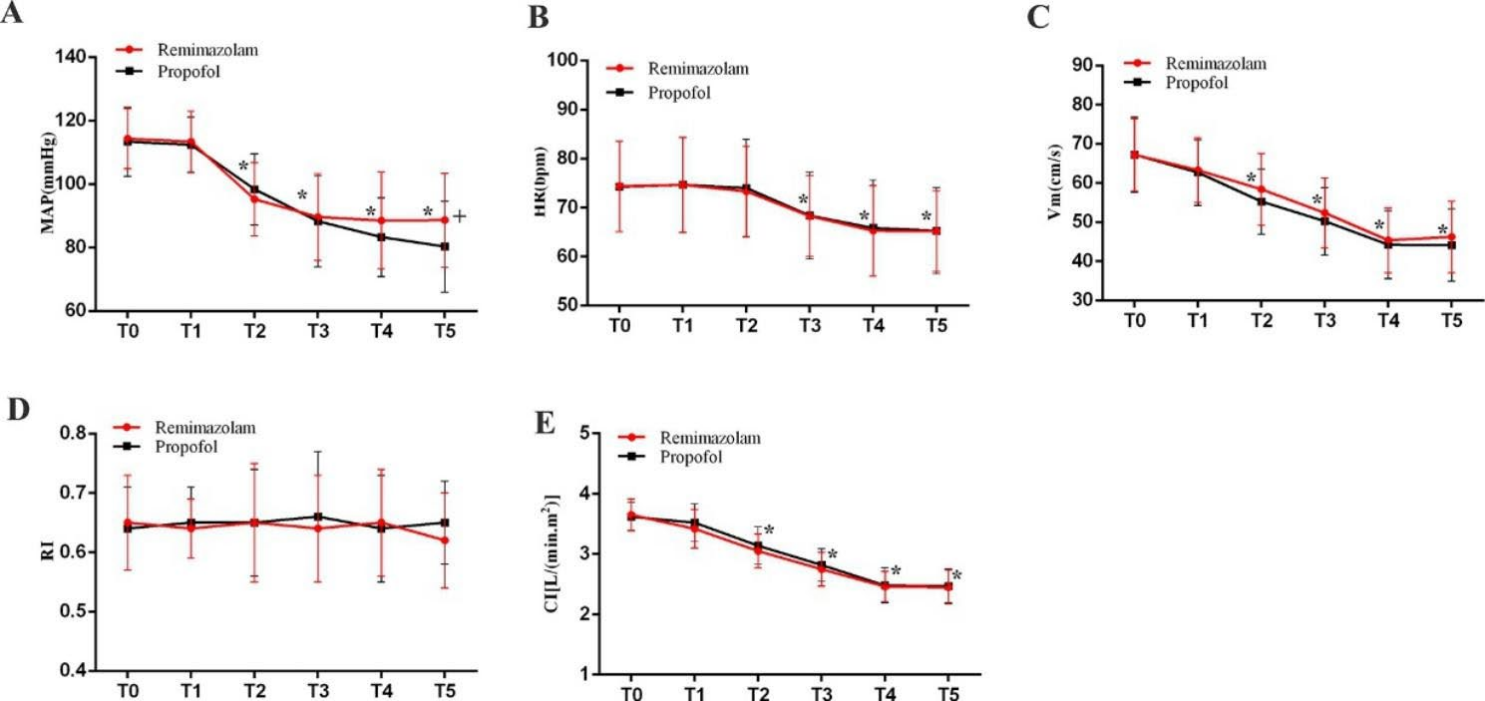
Hemodynamics index changes from T0 to T5. Changes in (**A**) mean arterial pressure (MAP), (**B**) heart rate (HR), (**C**) mean flow velocity (Vm), (**D**) resistance index (RI), (**E**) cardiac index (CI) from pre-induction of anesthesia until before intubation. Data are expressed as mean ± standard deviation. *P < 0.05 vs. Baseline in each group. + *P* < 0.05 vs. between two groups

CBF index including RI and Vm were similar from induction to pre-intubation and there were no significant differences between the remimazolam group and propofol group (Fig. 3). Vm showed a downward trend from T1-T5 in each group while RI appeared to be steady at each time point.

### Hemodynamics index in groups

The changes in hemodynamics index including MAP, HR and CI were consistent which showed a downward trend from T1-T5 in remimazolam group and propofol group. And there was no difference of HR or CI at each time point in two groups. It was worth noting that MAP of P group was lower than that of R group(*P* < 0.05) at T5(Fig. 3).

### BIS value in groups

**Fig. 4:**
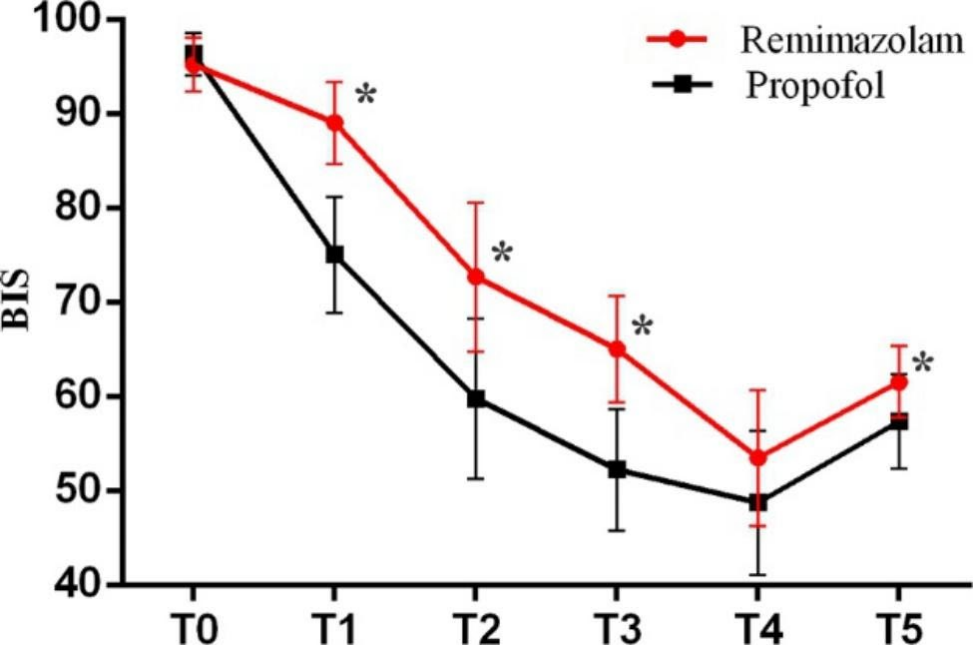
BIS value changes at post-induction of anesthesia(T1), loss of consciousness(T2), every 1 min for 2 min after loss of consciousness(T3-T4) and pre-intubation(T5). Data are expressed as mean ± standard deviation. *P < 0.05 vs. between two groups

The changes in BIS value showed a downward trend from T0-T4 in remimazolam group and propofol group, and both rise in reflex at T5. The statistics show that the BIS value of P group was lower than that of R group(*P* < 0.05) at T1-3 and T5, but there was no difference at T4 (Fig. 4).

## Discussion

Cerebral oxygen detector was used to detect the cerebral oxygen saturation of patients, which is a non-invasive real-time detection to analyze the oxygen supply level of patients’ brain tissue [16, 17]. Relevant studies have shown that the long-term low SrO_2_ level is closely related to the occurrence of postoperative adverse reactions, neurological recovery, length of hospital stay and mortality [18]. Therefore, SrO_2_ is often used in clinical anesthesia as a regulation and detection indicator to evaluate critically ill patients, extracorporeal circulation surgery and cerebrovascular disease surgery, predicting the prognosis of patients and preventing the occurrence of adverse events through this indicator [19].

In our study, remimazolam was noninferior compared with propofol in terms of oxygen saturation. SrO_2_ increased rapidly during induction and then slowly returned to baseline while preparing for endotracheal intubation. This may be accompanied by increased oxygen supply due to ventilation through the mask and decreased cerebral oxygen metabolism due to anesthesia drugs. Besides, mean and minimal SrO_2_ were similar between both groups but average values of minimal SrO_2_ were lower in P group than that in R group, which may be relevant with the reduction of cardiac output caused by propofol.

Decreased cardiac output during induction of general anesthesia or imbalance of cerebral perfusion pressure/cerebrovascular resistance may be the reason of decreased cerebral perfusion and thus decreased cerebral oxygen supply. The results of this study showed that the levels of Vm, CI and HR decreased during anesthesia induction, reached the minimum value before intubation in both groups. However, it was noted that propofol has a greater influence on patients’ blood pressure, leading to a larger decrease in MAP in group P after induction. This result suggests that, compared with propofol, remimazolam may have more advantages in hemodynamics of patients as anesthesia inducing drug, and this result is basically consistent with previous research results [20, 21].

However, the vascular resistance index RI remained basically stable at each time point, with no statistically significant change amplitude or difference between the two groups. We consider that the patients in this study are all elderly patients with poor regulation ability of their own blood vessels, and severe internal carotid artery stenosis induces blood–brain barrier injury and impairs the autoregulation to interrupt vasodilation or vasoconstriction [22–24].

Data from the research showed that after the induction of general anesthesia, the BIS value of P group was lower than that of R group due to the effect of remimazolam was slowly [25], and BIS values of two groups were similar until 2 min after loss of consciousness, indicating that remimazolam has similar sedative effect as propofol during the induction of general anesthesia.

To our knowledge, this is the first study applying remimazolam for the induction of anesthesia on carotid endarterectomy. We found that remimazolam-sufentanil anesthesia induction was comparable with propofol-sufentanil anesthesia induction in an aspect of preserving the SrO_2_ and maintaining hemodynamics in patients undergoing carotid endarterectomy. Furthermore, due to the small sample size and the lack of preoperative and postoperative follow-up in this study, more samples should be included and further research is required.

## Conclusions

Remimazolam-sufentanil anesthesia induction was comparable with propofol-sufentanil anesthesia induction in an aspect of preserving the SrO_2_ and stabilizing hemodynamics in patients undergoing carotid endarterectomy.

## Data Availability

The datasets analysed during the current study are available from the corresponding author upon reasonable request.

## Abbreviations

CEA: Carotid endarterectomy
SrO_2_: Cerebral oxygen saturation
Vm: Average blood flow velocity
RI: Resistance index
MAP: Mean arterial pressure
HR: Heart rate
CI: Cardiac index
CBF: Cerebral blood flow
CMRO_2_: Cerebral metabolic rate of oxygen
